# Histology and immune impact in OSCC

**DOI:** 10.1590/0103-644020267030

**Published:** 2026-07-24

**Authors:** Andressa Fernanda Paza Miguel, Nicole Lonni, Túlio Silva Rosa, Daniella Serafin Couto Vieira, Gustavo Davi Rabelo, Elena Riet Correa Rivero

**Affiliations:** 1 Postgraduate Program in Dentistry, Health Sciences Center, Federal University of Santa Catarina, Florianopolis, SC, Brazil.; 2 Department of Pathology, Health Sciences Center, Federal University of Santa Catarina, Florianopolis, SC, Brazil.

**Keywords:** Oral squamous cell carcinoma, tumour budding, perineural invasion, pattern of invasion, depth of invasio.

## Abstract

This study investigated how tumour histopathological characteristics and immune cell phenotypes in the tumour microenvironment influence the prognosis of patients with oral squamous cell carcinoma. Thirty-one samples of oral squamous cell carcinoma were analysed using immunohistochemistry to assess tumour budding, perineural invasion, depth of invasion, tumour thickness, lymphovascular invasion, pattern of invasion, and tumour-stroma ratio. Anti-CD66b and anti-CD8 immunostaining were used to examine neutrophil and lymphocyte infiltration within the tumour core, invasive front, and overall tumour. Kaplan-Meier survival curves were employed to correlate these features with patient survival. High tumour budding was observed in lesions with perineural and lymphovascular invasion and was positively associated with tumour thickness. Dispersed tumours had the highest depth of invasion and thickness, while non-cohesive tumours exhibited more perineural invasion and greater CD66b+ cell infiltration at the invasive front. Increased CD66b+ counts, non-cohesive and dispersed patterns, were associated with shorter overall survival. A higher ratio of neutrophils to lymphocytes was observed in patients who died. In conclusion, high tumour budding, perineural and lymphovascular invasion, and increased CD66b+ infiltration at the invasive front are linked to aggressive tumour behaviour and poor prognosis. These features may serve as important predictors of patient outcomes.

## Resumo

Este estudo investigou como as características histopatológicas tumorais e os fenótipos de células imunes no microambiente tumoral influenciam o prognóstico de pacientes com carcinoma epidermoide de boca. Trinta e uma amostras de carcinoma epidermoide de boca foram analisadas por imuno-histoquímica para avaliar brotamento tumoral, invasão perineural, profundidade de invasão, espessura tumoral, invasão linfovascular, padrão de invasão e a razão tumour-stroma. As imunorreações com anti-CD66b e anti-CD8 foram utilizadas para examinar a infiltração de neutrófilos e linfócitos no centro do tumor, na frente invasiva e na área tumoral como um todo. Curvas de sobrevida de Kaplan-Meier foram empregadas para correlacionar essas características com a sobrevida dos pacientes. Alto brotamento tumoral foi observado em lesões com invasão perineural e linfovascular, estando positivamente associado à espessura tumoral. Tumores com padrão disperso apresentaram maior profundidade de invasão e espessura, enquanto tumores não coesos exibiram maior invasão perineural e maior infiltração de células CD66b+ na frente invasiva. Contagens elevadas de CD66b+, padrões não coesivos e dispersos estiveram associados à menor sobrevida global. Uma maior razão entre neutrófilos e linfócitos foi observada em pacientes que foram a óbito. Em conclusão, alto brotamento tumoral, invasão perineural e linfovascular, e aumento da infiltração de CD66b+ na frente invasiva estão relacionados a comportamento tumoral agressivo e pior prognóstico. Essas características podem servir como importantes preditores de desfechos clínicos.

**Figure:**
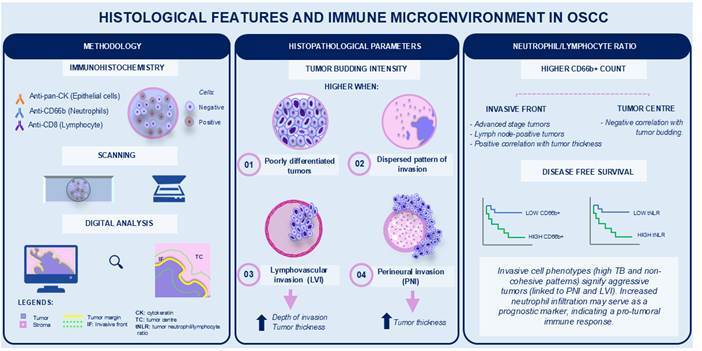

## Introduction

The incidence of lip and oral cavity cancers is projected to increase by 64.7% between 2022 and 2050, with a rising number of cases anticipated across all age groups and sexes, highlighting a significant global health burden ^1^. Risk assessment for oral squamous cell carcinoma (OSCC) relies on the tumour-node-metastasis (TNM) staging system ^2^ ^,^ ^3^. The latest edition of this system incorporates depth of invasion (DOI) and extracapsular spread in T and N classifications, respectively, due to their established prognostic value in OSCC survival outcomes ^2^ ^,^ ^3^. 

DOI is histologically measured from the basement membrane of the closest normal mucosa to the deepest point of tumour infiltration ^2^ ^,^ ^4^ ^,^ ^5^. DOI has been identified as a significant prognostic factor for overall survival (OS), disease-specific survival (DSS), and disease-free survival (DFS) in OSCC, with a prognostic cutoff of 4 to 5 mm ^2^ ^,^ ^4^. 

In addition to DOI and extracapsular spread, several other histopathological features are linked to OSCC survival outcomes, including perineural invasion (PNI), lymphovascular invasion (LVI), tumour budding (TB), the pattern of invasion (PI), and tumour-stroma ratio (TSR) ^4^. These features reflect underlying immune evasion mechanisms and inflammatory processes within the tumour microenvironment ^6^. 

Immune evasion is a hallmark of cancer progression ^6^. Within the tumour microenvironment, immune cells, particularly neutrophils and lymphocytes, play crucial roles in this process (7-9). Neutrophils can promote tumour growth and metastasis by releasing cytokines and growth factors, while lymphocytes, especially T cells, are essential for mounting an effective anti-tumour response. However, an imbalance, such as a high neutrophil-to-lymphocyte ratio (NLR), is frequently linked to immunosuppression and more aggressive histopathological features, which result in a poorer prognosis for cancer patients ^7^ ^,^ ^8^ ^,^ ^9^. 

In this context, the present study aims to evaluate TB, TSR, PNI, LVI, PI, and DOI using whole-slide imaging of digitized histological slides. Additionally, we quantified CD66b+ neutrophils and CD8+ T lymphocytes to assess the tumour neutrophil-to-lymphocyte ratio (tNLR), investigating the correlations among these parameters and determining their prognostic significance in patients with OSCC. The null hypothesis tested that the histopathological features and immune cell infiltrates assessed in this study are not significantly correlated with each other and do not hold prognostic value in patients with OSCC.

## Material and methods

### Study Population

Between 2013 and 2018, patients with histologically confirmed OSCC were treated at the University Hospital Professor Polydoro Ernani de São Thiago in Florianópolis, Brazil. Eligible cases involved primary OSCC treated with tumour resection, with follow-up data and adequate primary tumour material available for immunohistochemistry. A paraffin block was considered adequate if it contained a sufficient and well-preserved tissue sample, with minimal artefacts, clear tumour representation, and proper tissue orientation for immunohistochemical analysis. In cases with multiple blocks, the most representative block, demonstrating the highest tumour content and optimal tissue preservation, was selected. Patients with recurrent or secondary OSCC or prior chemoradiotherapy were excluded. A single examiner collected clinicopathological data, including age, sex, smoking and drinking habits, tumour site, TNM stage, histopathological grade, and follow-up information. The final sample included 31 cases of OSCC.

Follow-up data were retrieved from clinical records. The clinical endpoints were OS and DFS, defined as the time from the surgery date to the date of death or recurrence, respectively, or the last follow-up (April 2024). 

The institutional ethical committee review board approved this study (number 17674419.9.0000.0121), and the patients’ identities remained anonymous during data collection according to the Declaration of Helsinki.

### Immunohistochemical reactions and whole-slide image acquisition

Immunohistochemical reactions were performed using labeled polymer method (Agilent Cat# K4001, RRID:AB_2827819, Santa Clara, California, United States) to detect cytokeratin (1:50; Agilent Cat# GA053, RRID:AB_2892089, Santa Clara, California, United States), CD66b-positive neutrophils (1:500; BD Biosciences Cat# 555723, RRID:AB_396066, San Jose, California, United States), and CD8-positive T lymphocytes (ready-to-use; Agilent Cat# GA623, RRID:AB_3073940, Santa Clara, California, United States). Histological slides were scanned on Axio Scan.Z1 (RRID:SCR_020927, Carl Zeiss Microscopy GmbH, Jena, Thuringia, Germany) at 40x magnification (0.11 µm/pixel), and the resulting images were imported into QuPath software (RRID: SCR_018257, QuPath, Queen's University Belfast, Belfast, Northern Ireland, United Kingdom) for analysis. All histological evaluations were performed blinded to the clinical features.

Initially, to enhance stain separation, the ‘estimate stain vector’ command was applied to each image, utilising colour deconvolution in QuPath. Cytokeratin (CK)-immunostained slides were used to mark tumour borders with the ‘polygon’ tool. The invasive margin was determined by expanding the tumour borders by 500 µm, and the invasive front (IF) was delineated as a 1 mm wide area along the tumour border. The remaining region was designated as the tumour core (TC) ^8^. 

### Tumour budding

TB was defined as a single cell or a cluster of up to four tumour cells ^10^. Two examiners independently counted the number of TB using the CK-immunostained images. Calibration was performed by double counting 15 randomly selected cases with a 21-day interval (inter-observer agreement, 0.966). Subsequently, one observer performed TB count on the remaining sample (intra-observer agreement, 0.932). The highest TB area (hotspot) was identified at the IF. For analysis, a 0.785 mm^2^ area was drawn using the rectangle tool^10^, TB was manually counted using the “points” tool, and the software automatically generated the total number of buds. Samples were grouped using a two-tier system: low budding (< 5) and high budding (≥ 5) ^11^. 

### Tumour-stroma ratio

The tumour-stroma ratio (TSR) was assessed across the entire tumour area using CK-immunostained images. The “pixel classification” tool was used to visually determine thresholds for 3,3′-diaminobenzidine (DAB) and hematoxylin staining. Tumour-stroma areas were automatically calculated, and the TSR was determined by dividing the tumour area by the stromal area. A TSR value above 1 indicated tumour predominance (low stroma), while values below 1 showed a stromal predominance (high stroma) ^12^. 

### Histopathological characteristics: DOI, PNI, LVI, and PI

Two examiners independently assessed the histopathological features of H&E and CK-stained images. Criteria were defined during a calibration meeting; after which the assessments were cross-checked. In cases of disagreement, a third examiner was consulted to reach a consensus. 

DOI was measured as the perpendicular distance from the basement membrane of the closest normal mucosa to the deepest point of tumour invasion. Tumour thickness (TT) was calculated as the perpendicular distance from the highest point of the tumour surface to the deepest point of invasion ^13^. Both TT and DOI were categorised as <5 mm, ≥5 mm and <10 mm, and ≥10 mm. 

Perineural invasion (PNI) was identified when tumour cells infiltrated through the nerve sheath or encircled at least one-third of the nerve ^14^. Lymphovascular invasion (LVI) was defined as the unequivocal presence of tumour cells within the blood or lymphatic vessels, either lining the endothelium or focally adhered to the vascular wall ^15^. 

The PI was assessed at the IF and the worst area was considered and classified as i) cohesive, broad sheets of cancer cells and/or tumour nests with more than 15 cells across; ii) non-cohesive, narrow strands or small groups with fewer than 15 tumour cells, or single infiltrating tumour cells; iii) dispersed pattern, individual cells infiltrating at a distance of 1 mm from the main tumour ^15^. 

Semi-automated counting of immune cells and neutrophil to lymphocyte ratio (NLR).

CD8 and CD66b positive cells were assessed using the “fast cell count” command for the entire tumour (WT), TC, and IF. Positive cells were detected by separating stains using colour deconvolution and identifying peaks in the sum of the hematoxylin and DAB channels. The reliability of this method has been previously tested against manual counting, demonstrating excellent agreement between the two approaches (Supplemental material 1.1, detailed methodology). The results are presented as positive cells/mm^2^ of the tumour area. The tNLR was calculated by dividing the number of CD66b+ cells/mm^2^ by the number of CD8+ cells/mm^2^ for each region. 

### Statistical analysis

The Gaussian distribution of continuous variables was verified using the Shapiro-Wilk test. Normally distributed variables were described as means and standard deviation (SD), and comparisons between groups were conducted using Student’s t-test (two groups) and ANOVA (three groups). Mean differences and 95% confidence intervals (95% CI) were presented. Non-normally distributed variables were described using the median and interquartile range (IQR), with comparisons made with the Mann-Whitney test (2 groups) and Kruskal-Wallis test (3 groups). The calculation of effect size (ES) and the corresponding cutoff points are detailed in Supplemental material 1.2. The correlation between two continuous variables was tested using the Pearson correlation coefficient (r, normal distribution) and Spearman rank correlation coefficient (r_s_, non-normal distribution). Categorical variables were described as percentages, and associations were tested using Fisher’s exact test. Survival curves were generated using Kaplan-Meier analysis, with the log-rank test employed for comparisons. For the survival analysis, CD66b+, CD8+, and tNLR were dichotomised as high and low based on the median values. The data were analyzed using SPSS Statistics® (RRID:SCR_002865). Statistical significance was set at P < 0.05.

## Results

### Clinical features

The final sample included 31 patients (Supplemental material 2, Table 2S) with a mean age of 56.87 ± 9.39 years. The majority of patients were male (77.4%), smokers (87.1%), alcohol users (76.7%), and frequently diagnosed at an early stage (58%). Adjuvant therapy was administered to 54.95% of the patients, 6 (27.3%) experienced recurrence, and 8 (40.0%) died. The median follow-up time was 104.4 months (IQR: 97.3), ranging from 10 to 148.7 months. 

### Histopathological characteristics

Most tumours were classified as well-or moderately differentiated (90.3%). Twenty-three cases had identifiable adjacent normal mucosa, allowing for DOI measurement, while TT was assessed for the entire sample. The mean DOI was 5.84 mm ± 3.81 mm, and the mean TT was 6.5 mm ± 3.82 mm, resulting in a mean difference (DOI-TT) of -0.67 mm ± 0.91 mm. In most cases (86.95%), the DOI was lower than that of TT. Furthermore, the DOI was less than 5 mm in eight cases, between 5 and 10 mm in 12, and 10 mm or more in three. TT was less than 5 mm in 10 cases, between 5 and 10 mm in 14, and ≥ 10 mm in 7.

**Figure 1:**
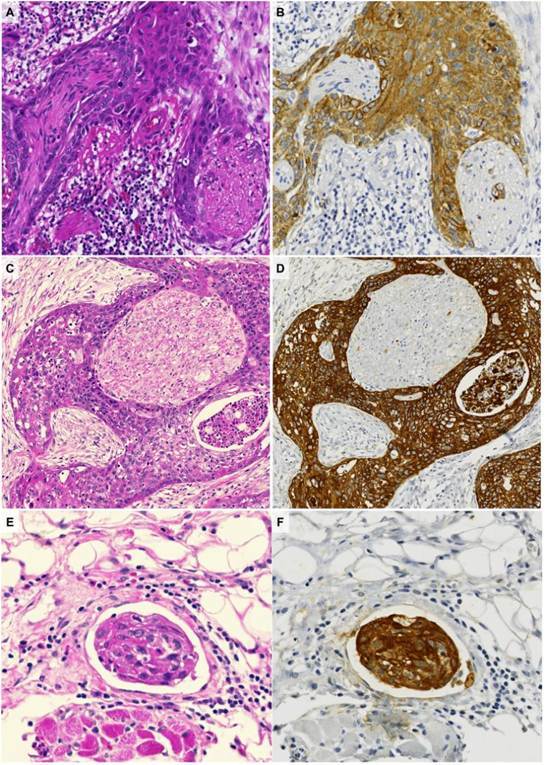
(A-D) Perineural invasion. (A) Hematoxylin and eosin-stained image showing partial encirclement of the nerve sheath by tumour cells. (B) The same region seen in (A) stained with cytokeratin and showing invasion of the nerve sheath by tumour cells. (C) Hematoxylin and eosin-stained and (D) cytokeratin-immunostained images showing complete encirclement of the nerve sheath by tumour cells. (E) Hematoxylin and eosin-stained and (F) cytokeratin-immunostained image showing tumour cells within the vascular space.

PNI was noted in 35.5% of cases, while LVI occurred in 25.8% (Figure 1). The predominant PI observed was non-cohesive (45.2%), followed by dispersed (38.7%), and cohesive (16.1%). Regarding DFS, cases with PNI had poorer outcomes (P=0.036). 

The median number of TB cases was 16 (IQR: 25) and the mean TSR was 0.7 ± 0.41. Well-differentiated tumours exhibited a lower number of TB than moderately differentiated tumours (P=0.002; r=0.59, large ES) and poorly differentiated tumours (P=0.011; r=0.75, large ES) (Table 1). The number of TB was higher in the dispersed pattern than in the cohesive (P=0.001; r=0.78; large ES) and non-cohesive patterns (P=0.029, r=0.43; medium ES). Tumours with LVI had a greater number of TB than those without (P=0.018, r=0.43; medium ES), and tumours with PNI also showed a higher number of TB than those without (P=0.002, r=0.56, large ES) (Table 1). 

**Table 1:** Mean (standard deviation) for tumour-stroma ratio (TSR), depth of invasion (DOI), tumour thickness (TT), and median (interquartile range) for tumour budding (TB), according to histopathological features.

		TSR	*P*	DOI	*P*	TT	*P*	TB	*P*
		Mean (SD)		Mean (SD)		Mean (SD)		Median (IQR)	
PI	Cohesive	0.83 (0.15)^a^	0.244	1.90 (3.03)^a^	0.042	2.37 (3.14)^a^	0.003	0.0 (1.0)^a^	0.001
	Non-cohesive	0.82 (0.46)^a^		5.91 (3.64)^ab^		6.59 (3.51)^ab^		16.0 (16.0)^a^	
	Dispersed	0.55 (0.34)^a^		7.51 (3.28)^b^		9.51 (3.95)^b^		32.0 (43.75)^b^	
PNI	No	0.70 (0.40)	0.97	4.94 (3.60)	0.089	5.32 (3.45)	0.001	8.5 (19.75)	0.002
	Yes	0.71 (0.46)		7.89 (3.74)		10.16 (4.05)		37.0 (46.0)	
LVI	No	0.67 (0.33)	0.506	5.50 (3.90)	0.369	6.46 (4.18)	0.210	11.0 (20.0)	0.018
	Yes	0.79 (0.59)		7.44 (3.37)		8.70 (4.51)		33.5 (47.5)	
HG	Well	0.56 (0.25)	0.185	4.58 (4.06)	0.247	5.63 (4.74)	0.466	5 (9)^a^	0.001
	Moderate	0.80 (0.44)		6.87 (3.71)		7.43 (3.80)		19 (27)^b^	
	Poor	0.40 (0.29)		3.02 (.65)		8.75 (6.61)		62 (39)^b^	

PI, pattern of invasion; PNI, perineural invasion; LVI, lymphovascular invasion; HG, Histopathological grade; TSR, tumour-stroma ratio; DOI, depth of invasion; TT, tumour thickness; TB, tumour budding; SD, standard deviation; IQR, interquartile range; P, P-value. TSR, DOI, and TT: Student’s t-test (two groups) and ANOVA test (three groups, post-hoc Tukey test). TB: Mann-Whitney test (two groups) and Kruskal-Wallis test (three groups). Statistical significance: P < 0.05. Different superscript letters indicate statistical differences between the groups.

DOI was higher in the dispersed pattern than in the cohesive (P=0.033; mean difference: 5.60; 95% CI: 1.34 - 9.85; Hedges’ g: 1.62 - very large ES), as was TT (P=0.003; mean difference: 7.14; 95% CI: 2.87 - .39; Hedges’ g: 1.81 - very large ES). TT was also higher in tumours with PNI than in tumours without (P=0.001; mean difference: 4.84; 95% CI: 2.02 - 7.65; Hedges’ g: 1.28 - very large ES) (Table 1). There was an association between PI and PNI, in which all cases with a cohesive pattern had no PNI, and the dispersed pattern frequently exhibited PNI (OR: 18.00; 95% CI: 2.47 - 131.28; P=0.004) (Table 2). 

**Table 2:** Perineural invasion and lymphovascular invasion according to the pattern of invasion, and lymphovascular invasion according to perineural invasion. Results are expressed as number (Percentage).

		PNI		*P*	LVI		*P*
		No	Yes		No	Yes	
PI	Cohesive	5 (100.0)	0 (0)	0.001	5 (100.0)	0 (0)	0.256
	Non-cohesive	12 (85.7)	2 (14.3)		11 (78.6)	3 (21.4)	
	Dispersed	3 (25.0)	9 (75.0)		7 (58.3)	5 (41.7)	
		LVI		*P*			
		No	Yes				
PNI	No	17 (85.0)	3 (15.0)				
Yes	6 (54.5)	5 (45.5)	0.095				

PI, pattern of invasion; PNI, perineural invasion; LVI, lymphovascular invasion; *P*, P value. Fisher’s exact test. Statistical significance: P<0.05.

There was a strong positive correlation between DOI and TT (r=0.971; P=0.000), and a moderate positive correlation between TT and TB (r_s_=0.407; P=0.023). 

### Immune cells and tumour NLR

When analysed in relation to clinical features, advanced-stage and lymph node-positive tumours exhibited a higher number of CD66b+ cells and a higher tNLR. In contrast, CD8+ cell counts were lower in these tumours, although this difference was not statistically significant (Supplemental material 3, Table 3S). 

**Figure 2:**
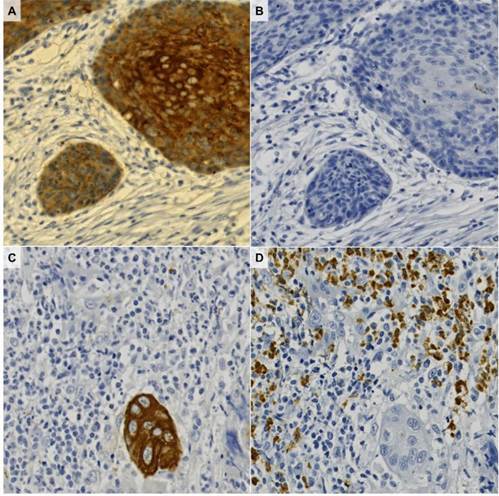
Tumour neutrophil: (A) Cytokeratin-stained and (B) CD66b-stained images showing low CD66b+ cell infiltration at the invasive front of cohesive tumours. (C) Cytokeratin-stained and (D) CD66b-stained images showing high CD66b+ cell infiltration at the invasive front of non-cohesive tumours.

The number of CD66b+ cells at IF was significantly higher in tumours with a non-cohesive pattern (median: 75.55, IQR: 281.43) compared to those with a cohesive pattern (median: 6.76, IQR: 38.39; P=0.025, r =0.52, large effect size) (Figure 2).

A moderate positive correlation was observed between CD66b+ cells at the IF and TT (r_s_: 0.386; P=0.032), as well between CD66b+ cells throughout the entire tumour and TT (r_s_: 0.345; P=0.057). There was a moderate negative correlation between CD66b+ cells at TC and TB (r_s_: -0.453; P=0.023) and between tNLR at TC and TB (r_s_: -0.493; P=0.012).

**Figure 3:**
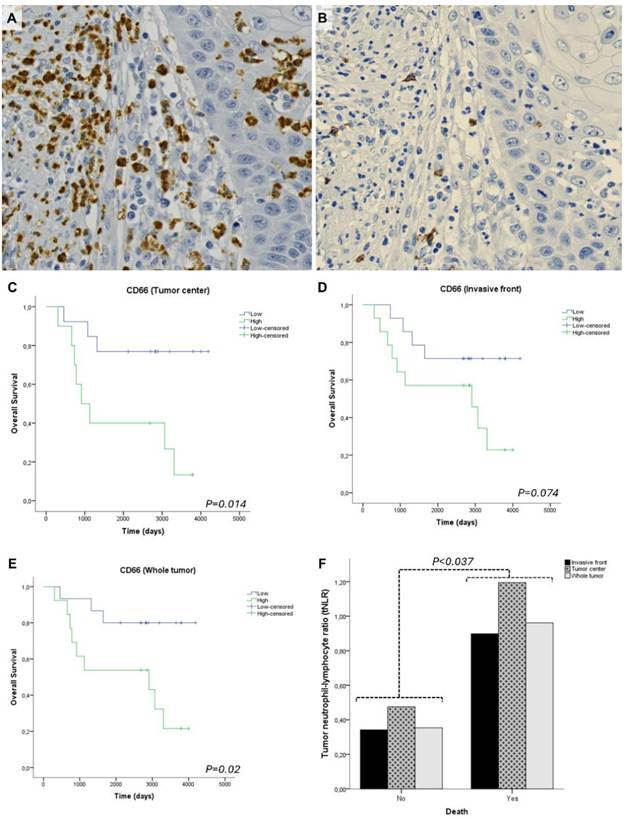
Tumour neutrophil and lymphocyte: (A) CD66b-immunostained and (B) CD8-immunostained images from the same tumour core region, showing high CD66b+ and low CD8+ cell densities (cells/mm²). Kaplan-Meier survival curves for overall survival, comparing high versus low CD66b+ counts (cutoff: median) at the (C) tumour core, (D) invasive front, and (E) whole tumour. (F) Mann-Whitney test comparing high versus low tumour neutrophil-lymphocyte ratio (tNLR) across all compartments, based on patient outcomes.

Elevated CD66+ cell counts (IF, TC, and WT) were observed in patients who did not survive (P<0.00001). In the OS analysis, patients with elevated CD66b+ counts at IF and WT experienced significantly shorter survival times than those with lower counts (P<0.049). Similarly, patients who died had elevated total tNLR values (P=0.043) (Figure 3).

No association was detected between CD8+ levels and the outcomes of interest. Additionally, none of the variables examined were associated with DFS.

## Discussion

The risk assessment OSCC traditionally relies on the TNM staging system. However, concerns about the effectiveness of TNM classification have emerged in recent years, as it fails to fully capture the complexity of tumour behaviour and prognosis ^2^. In line with these concerns, the 5th Edition of the World Health Organization (WHO) Classification of Head and Neck Tumours recommends integrating additional histological parameters into OSCC risk assessment ^2^. These adverse prognostic factors include DOI, TB, PNI, LVI, PI and TRS ^4^ ^,^ ^10^ ^,^ ^13^ ^,^ ^15^. In this way, the present study explored some histological criteria focusing on their potential relationship with the biological behaviour of OSCC, thereby addressing gaps left by the TNM system alone.

Both DOI and TT quantify the vertical extent of a primary tumour, yet they employ different reference points. DOI is measured from the basement membrane adjacent to the tumour, whereas TT is calculated from the tumour’s surface to the deepest malignant cells ^13^. In the present study, DOI was generally lower than the TT, with the difference being less than 1 mm in most cases. If we had categorized the sample using the 5 mm cutoff point, only one case would have changed the categories, with DOI < 5 mm and TT > 5 mm^14^. These results are consistent with those reported previously where DOI was usually lower than TT, and as T category modulators, the use of TT instead of DOI changed the T category in 5.7% and the clinical stage in 3.9% of cases, always by one category ^5^ ^,^ ^13^. Additionally, DSS analysis showed that risk stratification was similar when using DOI or TT as T-category modulators ^5^ ^,^ ^13^. In our study, DOI and TT levels were not associated with DFS; however, they were associated with high-risk histopathological features. DOI and TT were elevated in dispersed tumours, whereas TT was also higher in tumours with PNI and showed a moderate correlation with TB count. 

Although our sample predominantly featured low-risk clinical characteristics, we frequently observed high-risk histopathological features, such as a non-cohesive/dispersed PI, a high TB count, and a low TSR. The pattern of invasion was selected because it reflects tumour-stroma interaction and invasive dynamics that are not fully captured by conventional histological grading. While multi-tier invasion classifications are also used ^16^, their complexity and interobserver variability may limit routine applicability. The simplified patterns used here preserve biologically meaningful differences in invasive behaviour while improving reproducibility.

We also found that TB progressively increased from the cohesive to the dispersed PI, which was expected, as the criteria for classifying a tumour as non-cohesive or dispersed involves the presence of small clusters or individual tumour cells ^15^. TB has been suggested to be a histopathological marker for epithelial-mesenchymal transition (EMT) in carcinomas ^10^ ^,^ ^17^. Molecular changes related to EMT, such as increased expression of proteins associated with cell motility, such as vimentin, and loss of cell adhesion proteins, such as E-cadherin are observed in TB cells ^10^ ^,^ ^17^. EMT is the primary process through which malignant epithelial cells acquire an invasive phenotype, leading to invasion and metastasis ^10^ ^,^ ^17^. TB has been associated with worse OS, DSS, and DFS ^4^ ^,^ ^10^ ^,^ ^18^. In this study, although TB was not directly associated with survival outcomes, it was higher in cases exhibiting LVI and PNI, which are also high-risk histopathological features for poor survival ^4^ ^,^ ^19^ ^,^ ^20^.

In this study, we observed that PNI occurred in 35% of the cases and was related to shorter DFS. Schmidt et al. reported a similar prevalence of 42% when using immunohistochemistry to identify PNI ^21^. However, the prevalence of PNI identified with HE is lower, varying between 17-26% ^21^. OSCC exhibits a particular affinity for nerve involvement, and rates of PNI may go as high as 82% ^20^ ^,^ ^21^. PNI is a prognostic factor for poor survival ^20^ ^,^ ^21^. The molecular mechanism of nerve invasion results from the interaction between tumour cells and nerve components, especially Schwann cells. Tumour cells initiate communication with the nerve before coming into direct contact with it and can induce transcriptome changes in nerves in a distance-dependent manner ^22^. In addition, malignant cells from PNI-positive tumours express neurotrophic proteins such as nerve growth factor and its receptor tropomyosin receptor kinase A, which act as chemoattractants for cancer cells by triggering EMT changes, thus promoting cell invasiveness and facilitating PNI ^22^. 

The granulocyte marker CD66b, which is normally stored in cytoplasmic granules, becomes exposed upon neutrophil degranulation, serving as an indicator of neutrophil activation under pro-inflammatory stimuli ^23^. Increasing evidence highlights that neutrophils, particularly activated subsets, play a pivotal role in tumour progression and immune evasion. In head and neck squamous cell carcinoma, neutrophil infiltration has been consistently associated with poor OS, DFS and lymph node metastasis ^24^ ^,^ ^25^. In the present study, a high CD66b+ count at TC and WT was associated with worse OS. Zhou et al. conducted a similar analysis, distinguishing between the tumour core and invasive margin ^26^. Contrary our findings, the authors reported no significant association between DFS and CD66b+, and instead found an association between high CD8+ count at the TC and improved OS and DFS ^26^. Despite extensive research, the role of neutrophils in OSCC prognosis remains controversial, with conflicting findings across different studies ^27^ ^,^ ^28^.

In our study, a high CD8+ count was generally observed in association with early-stage disease and negative lymph nodes status, though statistical significance was not reached. Furthermore, we observed that the tNLR was higher in patients who died. A previous systematic review linked the NLR with adverse survival in OSCC ^9^. Therefore, we suggest that the tNLR could potentially be used as a prognostic factor in OSCC. The tumour microenvironment plays a crucial role in regulating immune responses, with immunosuppressive factors inducing a pro-tumoural phenotype in neutrophils while suppressing the activity of effector cells such as CD8+ lymphocytes ^28^. Hence, the tumour tNLR may reflect the balance between pro-tumoural and anti-tumoural immune responses ^29^. 

In this investigation, the invasive front of non-cohesive tumours exhibited higher CD66b+ cell infiltration than that of cohesive counterparts. This observation is consistent with previous work, who demonstrated that OSCC cell lines cultured in a neutrophil-conditioned medium exhibited increased migration and invasiveness ^29^. Additionally, these OSCC cells adopted a mesenchymal morphology, increased expression of vimentin, metalloproteinase 9, N-cadherin, alongside a concomitant reduction in E-cadherin expression, suggesting the occurrence of EMT ^29^. This evidence supports the notion that pro-tumorigenic neutrophils may drive phenotypic shifts in tumor cells, histologically manifested as a non-cohesive PI. Moreover, TGF-β is recognized as a key regulator of both neutrophil polarization and EMT processes ^6^ ^,^ ^29^. Although TGF-β levels were not directly measured in the present study, the observed association between neutrophil infiltration, worse survival, and frequent TB raises the hypothesis that elevated TGF-β signaling, commonly reported in OSCC, could underlie these histological and immunological features ^29^. Future studies are warranted to investigate this potential mechanism. 

This study has several limitations, primarily related to its small sample size and retrospective design. Furthermore, the study was conducted with cases from a single regional location, which may compromise the external validity of our findings and limit the generalisability of the results to a broader population. 

In conclusion, high-risk histopathological features were prevalent in this study and often interrelated. The DOI did not significantly impact tumour measurement, although it was generally lower than TT. Histopathological markers of cell invasiveness, such as PI and TB, were associated with PNI and LVI, which are features previously linked to regional spread, survival, and recurrence in OSCC. Additionally, a high CD66b+ count was identified as a prognostic factor for worse OS, with higher CD66b+ counts observed at the IF of non-cohesive tumours. Our results emphasize the prognostic value of several histological characteristics in predicting poorer outcomes; however, such comprehensive data are rarely incorporated into pathological reports. Future research should focus on utilizing these metrics to assess their potential for predicting the need for more aggressive treatment in OSCC management.

## Data Availability

The research data are available upon request.

## Supplementary Material

1 Supplementary methodology

1.1 Validation of the method's reliability against manual counting

One examinator blinded to the clinical features performed the semi-automated count, after an initial calibration, where the intra-observer agreement was 0.98 for CD66b and 0.979 for CD8. The agreement between manual and semi-automated methods was tested on 208 randomly chosen 1mm^2^ fields from 46 oral squamous cell carcinoma samples. The agreement was considered excellent.

**Table 1S:** Agreement between manual and semi-automated methods.

	CD66B	CD8
Intraclass correlation coefficient	0.96	0.99
Sperman’s correlation coefficient	0.97	0.98
Average of differences	1.37	-1.25
Standard deviation	39.74	40.62
Upper limit	79.27	78.37
Lower limit	-76.53	-80.86

1.2 Effect size calculation

a) Effect size Mann-Whitney test (*r*):

*r* = z / √n

*z: provided by SPPS after Mann-Whitney’s test

n: group size

Formula for z:

$$Z^{*}=\frac{U-\frac{n_{1\;}x{\;n}_{2}}{2}-0,5}{\sqrt{n_{1}\;x{\;n}_{2}\;x\;\frac{\left(n_{1}+n_{2}+1\right)}{12}}}$$

Values of *r* and effect size conclusion:

<0,1: Irrisory

<0,3: Small

<0,5: Medium

>0,5: Large

a) Effect size for Student T-test (Hedge’s g):

Hedge’s g= Cohen’s d x (1 - 3 / 4 x [n1 + n2] -9)

Cohen’s d= (Median group 1 / Median group 2) / combined standard deviation

*n: sample size of the group

Hedge’s g values and effect size conclusion:

<0,1: Null

<0,2: Very small

<0,5: Small

<0,8 Medium

<1,2: Large>1,2: Very large

References:

1. Cohen J. Statistical power analysis for the behavioral sciences. 2nd ed. Hillsdale (NJ): Routledge; 1988.

2. Fritz CO, Morris PE, Richler JJ. Effect size estimates: current use, calculations, and interpretation. J Exp Psychol Gen. 2012;141^(1)^:2-18.

3. Kerby DS. The simple difference formula: an approach to teaching nonparametric correlation. Compr Psychol. 2014;3:11.IT.13.11.

## 2. Supplementary Table 2S

**Table 2S:** Clinical features of the sample.

Clinical features		N (%)
Age	<60	18 (58.1)
	>60	13 (41.9)
Sex	Male	24 (77.4)
	Female	7 (22.6)
Smoking	No	4 (12.9)
	Yes	27 (87.1)
Drinking	No	6 (20.0)
	Yes	23 (76.7)
Site	Tongue	15 (48.4)
	Floor of the mouth	6 (19.4)
	Other	10 (32.3)
Tumor size (T)*	T1	11 (35.48)
	T2	6 (29.03)
	T3	6 (19.36)
	T4	3 (9.68)
Lymph node Involvement (N)*	N-	20 (64.51)
	N+	5 (16.13)
Clinical stage*	I	8 (25.80)
	II	6 (19.36)
	III	3 (9.68)
	IV	7 (22.58)
Histopathological grade	Well	9 (29.0)
	Moderate	19 (61.3)
	Poor	3 (9.7)
Perineural invasion	No	20 (64.5)
	Yes	11 (35.5)
Lymphovascular invasion	No	23 (74.2)
	Yes	8 (25.8)
Pattern of invasion	Cohesive	5 (16.1)
	Non-cohesive	14 (45.2)
	Dispersed	12 (38.7)
Tumor budding	<5	4 (12.9)
	≥5	27 (87.1)
Tumor-stroma ratio	≥1 - low stroma	8 (25.8)
<1 - high stroma	23 (74.2)
Treatment	Resection	14 (45.2)
	Resection + RQT	7 (22.6)
	Resection + RT	10 (32.3)
Death	No	16 (55.2)
	Yes	13 (44.8)
Recurrence	No	19 (63.3)
	Yes	11 (36.7)

NI, not informed; RQT, radio-chemotherapy; RT, radiotherapy. * There were missing data on T stage (2 patients), N stage (6 patients), and clinical stage (7 patients).

## 3 Supplementary Table 3S

**Table 3S:** Median (interquartile range) of the positive tNLR, CD8/mm², CD66b/mm^2^ for each tumoral region, according to lymph node involvement.

	N STATUS	IF	P	TC	P	WT	*P*
		Median (IQR)		Median (IQR)		Median (IQR)	
tNLR	N-	0.08 (0.23)	0.243	0.14 (0.46)	0.559	0.10 (0.26)	0.243
	N+	0.24 (2.37)		0.41 (3.71)		0.36 (2.94)	
CD8+	N-	270.13 (265.95)	0.336	191.08 (325.28)	0.444	215.38 (274.63)	0.272
	N+	117.02 (661.97)		61.52 (442.43)		56.98 (508.83)	
CD66b+	N-	21.20 (91.47)	0.447	23.92 (78.41)	0.444	22.73 (45.53)	0.621
	N+	33.56 (161.60)		24.51 (167.39)		22.09 (153.97)	

IF, invasive front; TC, tumor core; WT, whole tumor; IQR, interquartile range; P, P value; N, lymph node involvement. Mann-Whitney test (2 groups). Statistical significance: P < 0,05.

## 4 Supplementary Table 4S

**Table 4S:** Results of the Kaplan-Meier analysis for overall survival.

Overall survival				
		Events (n)	95% confidence interval	P value
Stage	I/II	5	2478.74 - 3775.19	0.061
	III/IV	6	1215.32 - 3080.67	
PI	Cohesive	0	-	0.05
	Non-cohesive	6	-	
	Dispersed	7	-	
CD66b IF	<34.89	4	2618.58 - 4049.98	0.074
	≥34.89	9	1522.79 - 3033.68	
CD66b TC	<40.27	3	2694.16 - 4191.37	0.014
	≥40.27	8	986.78 - 2627.42	
CD66b WT	<23.84	3	2952.57 - 4208.75	0.020
	≥23.84	9	1438.88 - 2979.04	

PI, pattern of invasion; LVI, lymphovascular invasion; IF, invasive front; TC, tumor core; WT, whole tumor. Statistical difference set at P < 0,05 (Kaplan Meier, log rank test). Bold letters indicate statistical differences. - Statistics could not be calculated.
